# Differences of Enzymatic Activity During Composting and Vermicomposting of Sewage Sludge Mixed With Straw Pellets

**DOI:** 10.3389/fmicb.2021.801107

**Published:** 2022-01-10

**Authors:** Ales Hanc, Bayu Dume, Tereza Hrebeckova

**Affiliations:** Department of Agroenvironmental Chemistry and Plant Nutrition, Faculty of Agrobiology, Food and Natural Resources, Czech University of Life Sciences, Prague, Czechia

**Keywords:** enzymatic activity, sewage sludge, straw pellets, composting, vermicomposting, microorganisms, earthworms

## Abstract

The study aims were focused on profiling eight hydrolytic enzymes by fluorescence method using a multifunctional modular reader and studying the proportion of basic microorganism groups during composting and vermicomposting of sewage sludge mixed with straw pellets in several proportions (0, 25, 50, 75, and 100%). The greatest decrease in enzymatic activity occurred in the first half of composting and vermicomposting. After 4 months of these processes, the least enzymatic activity was observed in the sludge with 50% and also 25% straw addition, indicating that straw is an important means for the rapid production of mature compost from sewage sludge. Enzymatic activity was usually less in the presence of earthworms than in the control treatment because some processes took place in the digestive tract of the earthworm. For the same reason, we observed reduced enzyme activity during fresh feedstock vermicomposting than precomposted material. The final vermicompost from fresh feedstocks exhibited less microbial biomass, and few fungi and G− bacteria compared to precomposted feedstock. The enzymatic activity during composting and vermicomposting of sewage sludge and their mixtures stabilized at the following values: β-D-glucosidase—50 μmol MUFG/h/g dw, acid phosphatase—200 μmol MUFP/h/g dw, arylsulphatase—10 μmol MUFS/h/g dw, lipase—1,000 μmol MUFY/h/g dw, chitinase—50 μmol MUFN/h/g dw, cellobiohydrolase—20 μmol MUFC/h/g dw, alanine aminopeptidase—50 μmol AMCA/h/g dw, and leucine aminopeptidase—50 μmol AMCL/h/g dw. At these and lesser values, these final products can be considered mature and stable.

## Introduction

The global production of sewage sludge is estimated at 45 million tons per year [expressed in dry matter (Zhang et al., 2017)]. The most common methods of handling dewatered treated sludge include direct application to agricultural land, landfilling, and composting. In the Czechia, sludge production was 228 thousand tons of dry matter in 2018, i.e., 21 kg per capita. Approximately 1/3 of this amount was composted (Eurostat, 2021).

The course of the composting process and the physico-chemical and biological parameters of the final compost are especially affected by the addition of various bulking agents (Wang et al., 2021). Sludge composting with many bulking agents was previously described, e.g., sawdust, coffee husks, and brewery waste (Manga et al., 2021), yard trimming wastes (Robledo-Mahón et al., 2020), and cornstalks (Yuan et al., 2016). Composting is characterized by a thermophilic phase and the need to turn over or to aerate the composted material. Conversely, vermicomposting requires that the temperature does not exceed 35°C, in which case the earthworms die. The optimal temperature is between 20 and 25°C. Aeration is provided directly by earthworms by creating passages in the material. Earthworms act as mechanical blenders, and by disintegrating the organic matter, they change its physical and chemical properties, especially by gradually reducing the C:N ratio and increasing the surface area exposed to microorganisms. Thus, earthworms make feedstocks much more favorable for microbial activity and further decomposition (Dominguez and Edwards, 2011). At first, ingestion, digestion, assimilation, and the influence of earthworm gut microorganisms are involved in organic matter decomposition, and the processes associated with casting follow (Gómez-Brandón et al., 2011). Casting processes are more closely related to aging in maturation phase when the vermicompost is expected to reach optimum levels in terms of its biological parameters, thereby promoting plant growth and suppressing plant diseases. Little is known about determining the optimum levels (Domínguez and Gómez-Brandón, 2012). Degradation or utilization of substrate by microorganisms involves significant processes like nutrient cycling and transforming organic matter.

In the process, microbial activity could be assessed effectively with the analysis of enzymatic activity and microbial respiration. Soil enzyme activities provide information on the potential of soils to perform biochemical reactions (Błońska et al., 2017; Uzarowicz et al., 2020). Enzymatic activity was also explored as a possible tool for compost characterization and determination of compost maturity (Mondini et al., 2004). The biochemical reactions are brought about by enzyme catalytic action. Enzymes are divided into seven classes based on the reaction type that they catalyze, namely:

• Oxidoreductases—acting on the CH–OH group of donors, the aldehyde or oxo group of donors, CH–CH group of donors, CH–NH_2_ group of donors, CH–NH group of donors, etc.
• Transferases—transferring one-carbon groups, aldehyde, or ketonic groups, alkyl or aryl groups, other than methyl groups, nitrogenous groups, phosphorus-containing groups, etc.
• Hydrolases—acting on ester bonds, ether bonds, peptide bonds, carbon–nitrogen bonds, carbon–carbon bonds, phosphorus–nitrogen bonds, carbon–sulfur bonds, etc.
• Lyases—e.g., carbon–carbon lyases, carbon–oxygen lyases, carbon–nitrogen lyases, carbon–phosphorus lyases.
• Isomerases—e.g., racemases and epimerases, intramolecular isomerases and lyases.
• Ligases—forming carbon–oxygen bonds, carbon–sulfur bonds, carbon–nitrogen bonds, etc.
• Translocases—catalyzing the translocation of hydrons, inorganic cations, inorganic anions and their chelates, etc. (Nomenclature Committee of the International Union of Biochemistry and Molecular Biology [NC-IUBMB], 2021).

Some enzymes are important in organic material decomposition, organic matter transformation, nutrient cycling, nitrogen fixation, detoxification of hazardous substances such as xenobiotics, pesticides, pharmaceutical and personal care products, etc., and thus regulate the ecosystem (Gunjal et al., 2019). We studied eight hydrolase enzymes because they participate in metabolic processes during organic matter decomposition. These are as follows:

• β-glucosidase is widely distributed in nature and is related to the carbon cycle, acting in the cleavage of cellobiose into glucose molecules. Because of its sensitivity, this enzyme is considered as soil quality indicator and is directly related to the quantity and quality of soil organic matter. Furthermore, the addition of soil organic residues, such as biosolids, manure, urban sludge, and poultry litter, increases the activity of this enzyme in soil (Almeida et al., 2015). A long-term field experiment utilizing barley received four different treatments prior to sowing: municipal solid waste compost at either 20 t/ha (C20) or 80 t/ha (C80), cow manure at 20 t/ha (MA), and mineral fertilizer either NPK 400 kg/ha or NH_4_NO_3_ 150 kg/ha (MIN). The enzymatic activity of β-glucosidase was higher by 38, 62, 87, and 6% for C20, C80, MA, and MIN, respectively, compared with control unfertilized variant (García-Gil et al., 2000).
• Acid phosphatase—Phosphate-solubilizing micro- organisms are crucial for the transformation of organically bounded phosphorus into bioavailable forms by excreting extracellular phosphatase in the form of acid and alkaline phosphatase (Zheng et al., 2021).
• Arylsulphatase hydrolyzes aromatic sulfate esters and releases SO_4_^2–^. It is an indicator of sulfur mineralization in soils and also is important in the cycling of this element (Uzarowicz et al., 2020).
• Lipases are ubiquitous enzymes that catalyze the breakdown of fats and oils with the subsequent release of free fatty acids, diacylglycerols, monoglycerols, and glycerol. Besides this, they are also efficient in various reactions, such as esterification, transesterification, and aminolysis, in organic solvents. Therefore, those enzymes are nowadays extensively studied for their potential industrial applications. Examples in the literature concerning their use in different fields are numerous, such as resolution of racemic mixtures, synthesis of new surfactants and pharmaceuticals, oil and fat bioconversion, and detergency applications (Villeneuve et al., 2000).
• Chitinases are able to degrade the chitin chain. Chitinases are classified as exochitinases or endochitinases. Exochitinases cleave chitin from the open ends, while endochitinases cleave chitin at random positions. Fungal chitinases belong to the glycoside hydrolase family 18 and are divided into three phylogenetic groups—A, B, and C—and further subdivided into several subgroups. Chitinases are involved in different aspects of fungal biology, including fungal–fungal interactions, nutrient acquisition, cell wall remodeling, and autolysis (Tzelepis and Karlsson, 2021).
• Cellobiohydrolases are among the most important enzymes functioning in crystalline cellulose hydrolysis, significantly contributing to the efficient biorefining of recalcitrant lignocellulosic biomass into biofuels and bio-based products. Filamentous fungi are recognized as both well-known producers of cellulolytic enzyme commercial preparations and efficient hosts for heterologous protein secretion (Zoglowek et al., 2015).
• Aminopeptidases belong to exopeptidases, proteolytic enzymes that remove amino acids from the termini of peptides and proteins. They attack their substrates exclusively from the amino terminal end. Most remove one amino acid at a time, but a small group cleaves two or three residues. Alanine and leucine aminopeptidases release terminal nitrogen from amino acids (mainly from alanine and leucine), peptides, amides, and arylamides (Bradshaw, 2013).

Commercially derived hydrolytic enzymes are very expensive, whereas enzymes produced from mixed biosolids are very cheap and perform similarly to commercially produced enzymes (Nabarlatz et al., 2012). Enzymes secreted by microbial species or commercially produced enzymes were used in biosolid pretreatment for increasing methane production (Sethupathy et al., 2020).

In the literature, there are hydrolytic enzyme activity values, which were determined by classical colorimetric methods. We used a fluorescence method with multifunctional modular reader, which is a fast, modern, and economically advantageous method for determining the enzymatic activity in a large sample number. The enzymatic activity trends and values of eight hydrolytic enzymes identified in this study can be a guide for research and control institutes and for producers of composts and vermicomposts based on sewage sludge. The values can be used to predict compost and vermicompost maturity and to determine the time when the enzymatic activity no longer changes. The aims of the study were as follows: (i) to compare the enzymatic activity of eight hydrolytic enzymes and a proportion of microorganism groups during composting and vermicomposting of sewage sludge mixed with straw pellets, (ii) to find the impact of precomposting on enzymatic activity, microorganisms, and earthworm presence, (iii) to determine the effect of addition of straw pellets on the above-mentioned biological properties.

## Materials and Methods

### Feedstocks

Sewage sludge was obtained from a sewage treatment plant located in a small town in the Czechia. The sludge did not undergo any stabilization process. The dry matter content was 13.3%, and the total content of C, N, P, K, Ca, and Mg was 32.9, 5.3, 1.6, 0.5, 1.4, and 0.5% in dry matter, respectively. The pH/H_2_O (1:5, w/v) value was 7.0. The content of pollutants did not exceed the valid legislation. Pelletized straw pellets (PWS) were bought at the Granofyt Ltd., company. The pellet diameter was 10 mm. Because of very reduced pellet moisture, they were mixed with hot water (60°C) at the rate of 1:4 (w/v) before experimental use. The pellets were added to the sludge to plump the structure with enough air and an increased C:N ratio (53.2). The total content of C, N, P, K, Ca, and Mg was 42.6, 0.8, 0.1, 0.6, 0.4, and 0.1% in dry matter, respectively. The enzymatic activity and content of the main microorganism groups in feedstocks are shown in Table 1.

**TABLE 1:** Enzymatic activity and content of microorganisms in input raw materials.

	Sewage sludge	Peletized wheat straw
β-D-glucosidase (μmol MUFG/h/g dw)	679 ± 78	22 ± 3
Acid phosphatase (μmol MUFP/h/g dw)	4,411 ± 690	80 ± 3
Arylsulphatase (μmol MUFS/h/g dw)	173 ± 32	4 ± 0
Lipase (μmol MUFY/h/g dw)	9,020 ± 131	283 ± 31
Chitinase (μmol MUFN/h/g dw)	556 ± 81	1 ± 2
Cellobiohydrolase (μmol MUFC/h/g dw)	270 ± 2	4 ± 4
Alanine aminopeptidase (μmol AMCA/h/g dw)	1,185 ± 116	0 ± 0
Leucine aminopeptidase (μmol AMCL/h/g dw)	880 ± 67	2 ± 0
Fungi (μg PLFA/g dw)	146 ± 2	24 ± 3
Bacteria (μg PLFA/g dw)	3,150 ± 83	53 ± 6
Actinobacteria (μg PLFA/g dw)	34 ± 0	1 ± 0
G+ (μg PLFA/g dw)	1,159 ± 34	20 ± 3
G− (μg PLFA/g dw)	1,766 ± 47	22 ± 4
Total microbial biomass (μg PLFA/g dw)	4,145 ± 93	122 ± 9

*Values are means ± SD (n = 3). dw, dry weight; MUFG, 4-methylumbellyferyl-β-D-glucopyranoside; MUFP, 4-methylumbellyferyl-phosphate; MUFS, 4-methylumbellyferyl sulfate potassium salt; MUFY, 4-methylumbellyferyl-caprylate; MUFN, 4-methylumbellyferyl-N-acetylglucosaminide; MUFC, 4-methylumbellyferyl-N-cellobiopyranoside; AMCA, L-alanine-7-amido-4-methylcoumarin; AMCL, L-leucine-7-amido-4-methylcoumarin; PLFA, phospholipid fatty acids.*

### Experimental Design

#### Composting

For composting, five treatments were established:

(1) sludge 100%
(2) sludge 75% + PWS 25%
(3) sludge 50% + PWS 50%
(4) sludge 25% + PWS 75%
(5) PWS 100%

The feedstocks and its mixtures were thoroughly stirred and then were composted for 4 months in aerobic composters with 70-L working volume and 56-cm diameter. Aeration was provided from the bottom of the composter by a compressor. For the first 14 days (thermophilic phase), the air flow was set at 4 L/min for 5 min every half an hour and then for 3 min every half an hour. A temperature probe was inserted from the top of the composter to reach half the material height. The temperature was recorded every hour, and the values were collected in a data logger. Prior to sampling at the end of each month, any leachate was poured back into the composted material to achieve a closed loop of substances, and then the composted material was mixed thoroughly to achieve maximum homogeneity. Six samples (*n* = 6) of 0.5 kg each were taken.

#### Vermicomposting of Fresh and Precomposted Feedstock

For vermicomposting, five treatments were established as in the case of composting. Each treatment was carried out in triplicate without (as a control) and with earthworms as follows:

(1) sludge 100% without earthworms.
(2) sludge 100% with earthworms.
(3) sludge 75% + PWS 25% without earthworms.
(4) sludge 75% + PWS 25% with earthworms.
(5) sludge 50% + PWS 50% without earthworms.
(6) sludge 50% + PWS 50% with earthworms.
(7) sludge 25% + PWS 75% without earthworms.
(8) sludge 25% + PWS 75% with earthworms.
(9) PWS 100% without earthworms.
(10) PWS 100% with earthworms.

Fresh feedstocks were used in one set and precomposted feedstocks in the second set. Precomposting was conducted in composters, as described above, for 14 days. Vermicomposting trays measuring 40 × 40 × 15 cm were used for this part of the experiment. For vermicomposting with earthworms, 2/3 of the trays were filled with fresh or precomposted feedstocks (9 kg) and 1/3 with earthworm substrate based on grape marc (3 L). The bulk density of this fresh substrate was 685 g/L. The average earthworm density in the substrate was 125 pieces/L, with an average weight of 0.2 g per piece. The materials were separated by a mesh with 6-mm hole diameter. The earthworm substrate was utilized from the side to allow earthworms to move freely between materials in case of unsuitable conditions in the tested feedstocks, especially in case of ammonia formation in sewage sludge, which is toxic to earthworms. To ensure homogeneity, subsamples were taken from five sites in the tray (near the corners and in the middle). The material in the tray could not be mixed before sampling, as there would be a risk of death of the earthworms present. The total sample weight taken up at the end of each month was 0.5 kg from each of the three trays (*n* = 3).

### Sample Analyses

With respect to vermicomposting samples, earthworms were taken, counted, washed, and weighed. The cocoon number was also determined. To determine the enzymatic activity and the presence of microorganism groups, 150 g of the sample was frozen at −25°C, which was subsequently lyophilized.

For hydrolytic enzyme determination, a suspension was prepared by homogenizing 0.2 ± 0.002 g of a lyophilized compost or vermicompost sample and 20 ml of acetate buffer (pH = 5) at a concentration of 50 mmol L^–1^ for approximately 30 s using an Ultra-Turrax instrument (IKA Labortechnik, Germany). Then, 200 μl of the homogenized suspension was pipetted into a microtiter plate, and then the appropriate substrate at the given concentration (c) was added depending on the enzyme [β-D-glucosidase: 4-methylumbellyferyl-β-D-glucopyranoside (MUFG) at *c* = 2.75 mmol/L, acid phosphatase: 4-methylumbellyferyl-phosphate (MUFP) at *c* = 2.75 mmol/L, arylsulphatase: 4-methylumbellyferyl sulfate potassium salt (MUFS) at *c* = 2.50 mmol/L, lipase: 4-methylumbellyferyl-caprylate (MUFY) at *c* = 2.50 mmol/L, chitinase: 4-methylumbellyferyl-N-acetylglucosaminide (MUFN) at *c* = 1.00 mmol/L, cellobiohydrolase: 4-methylumbellyferyl-N-cellobiopyranoside (MUFC) at *c* = 2.50 mmol/L, alanine aminopeptidase: L-alanine-7-amido-4-methylcoumarin (AMCA) at *c* = 2.50 mmol/L, leucine aminopeptidase: L-leucine-7-amido-4-methylcoumarin (AMCL) at *c* = 2.50 mmol/L]. The microtiter plates were then placed in an incubator heated to 40°C for 5 min. Afterward, the substrate fluorescence was measured using a Tecan Infinite^®^ M200 (Tecan, Austria). Subsequently, the plates were again placed into an incubator for 2 h, and the fluorescence was measured again. From the difference between the initial and final value, the enzymatic activity was calculated and is usually given in micromoles of the respective substrate per hour and per gram of sample (Baldrian, 2009; Štursová and Baldrian, 2011). Fluorescence determination using a multifunctional modular reader is fast, modern, and an economically advantageous method for determining the enzymatic activity in a large sample number.

Samples for phospholipid fatty acid (PLFA) determination were extracted using phosphate buffer, chloroform, and methanol (0.8:1:2; v/v/v). Gas chromatography–mass spectrometry (450-GC, 240-MS Varian, Walnut Creek, CA, United States) was employed for determination of fatty acid methylated esters. The details of the analyses are described in Hanc et al. (2021).

A CHNS Vario MACRO cube analyzer (Elementar Analysensysteme GmbH, Germany) was used to determine total carbon and nitrogen *via* a thermal conductivity detector according to Hanc et al. (2017). The total contents of P, K, Ca, and Mg were determined by decomposition obtained by pressurized wet-ashing (HNO_3_ + H_2_O_2_) of dried samples in a closed system of Ethos 1 (MLS GmbH, Germany). Afterward, the element content was determined using ICP-OES (Agilent 720, Agilent Technologies Inc., United States) according to García-Sánchez et al. (2017).

### Statistical Analysis

Values are arithmetic means of three to six values (according to treatment) ± standard deviations. Based on the results of normality and homogeneity tests, the non-parametric Kruskal–Wallis test (*P* ≤ 0.05) was chosen for statistical analyses.

## Results

### Composting

**FIGURE 1:**
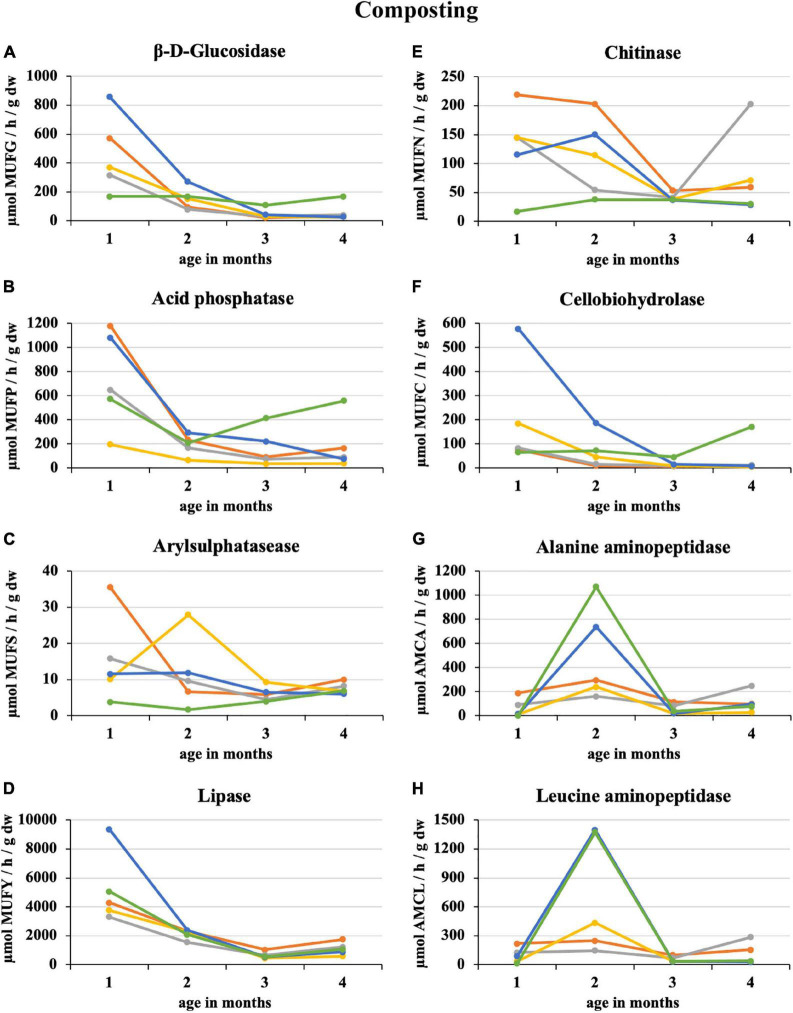
Course of enzymatic activity during composting of fresh feedstock. 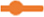, sludge 100%; 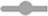, sludge 75% + PWS 25%; 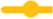, sludge 50% + PWS 50%; 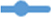, sludge 25% + PWS 75%, 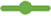, PWS 100%; PWS, pelletized wheat straw; dw, dry weight; MUFG, 4-methylumbellyferyl-β-D-glucopyranoside; MUFP, 4-methylumbellyferyl-phosphate; MUFS, 4-methylumbellyferyl sulfate potassium salt; MUFY, 4-methylumbellyferyl-caprylate; MUFN, 4-methylumbellyferyl-N-acetylglucosaminide; MUFC, 4-methylumbellyferyl-N-cellobiopyranoside; AMCA, L-alanine-7-amido-4-methylcoumarin; AMCL, L-leucine-7-amido-4-methylcoumarin.

After the first month of composting 100% sludge, there was a sharp 64% decrease in the enzymatic activity of the eight monitored enzymes. The smallest decrease by 16% was recorded for β-D-glucosidase, and the greatest by 84% was for alanine aminopeptidase. The least enzymatic activity at the composting end was recorded for 50% sludge (acid phosphatase, lipase, cellobiohydrolase, and alanine aminopeptidase) and 25% straw addition (β-D-glucosidase, arylsulphatase, chitinase, and leucin aminopeptidase) as illustrated in Figure 1. Conversely, the 100% straw was the least mature because of still ongoing decomposition characterized by a great activity of β-D-glucosidase, acid phosphatase, and cellobiohydrolase. Similarly, arylsulphatase and lipase increased in the second half of the composting period in the case of 100% sludge. The greatest enzymatic activity was recorded in the first month of composting, except for alanine and leucine aminopeptidase, which had their distinct peaks in the second month, especially in PWS 100% and sludge 25% + PWS 75%. For most enzymes, a statistically significant difference was found between the first and second halves of the composting process. The statistical differences among the treatments varied according to the individual enzymes and on a time course basis.

**FIGURE 2:**
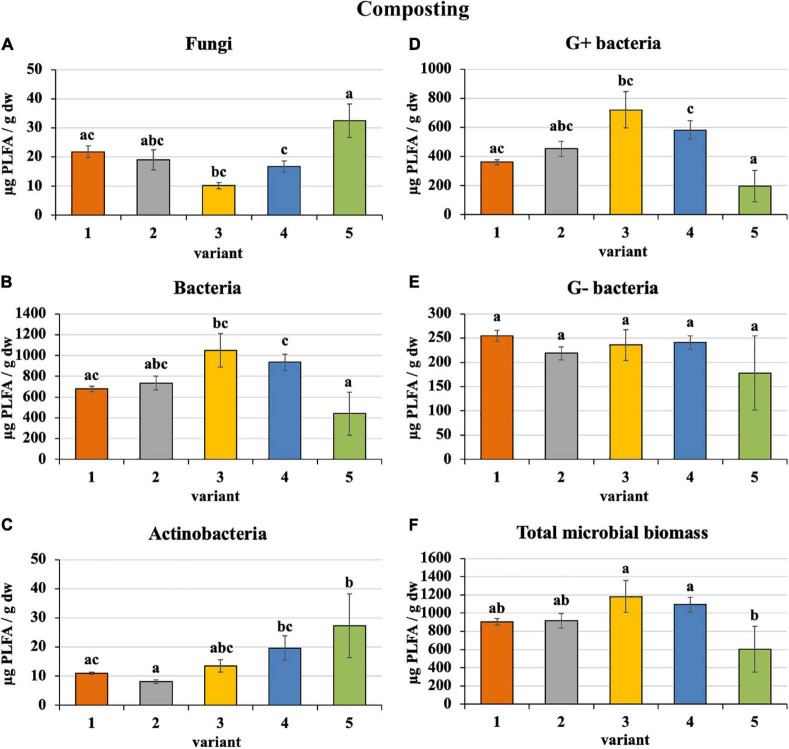
Content of microorganisms after composting feedstock. Treatments: (1) sludge 100%, (2) sludge 75% + PWS 25%, (3) sludge 50% + PWS 50%, (4) sludge 25% + PWS 75%, and (5) PWS 100%. Values are means ± SD (*n* = 3). Different letters indicate significant differences among treatments (Kruskal–Wallis test, *P* ≤ 0.05). PWS, pelletized wheat straw; PLFA, phospholipid fatty acids; dw, dry weight.

Sewage sludge 100% differed from PWS 100% in the content and proportion of microorganism groups expressed by PLFA. During composting of 100% sewage sludge, total microbial biomass, fungi, bacteria, actinobacteria, and G+ and G− bacteria decreased and accounted for 22, 15, 22, 32, 31, and 14% of used feedstock, respectively. Conversely, microorganism content increased in PWS 100% at the end with respect to total microbial biomass, fungi, bacteria, actinobacteria, and G+ and G− bacteria by 4. 9-, 1. 4-, 8. 3-, 27. 3-, 9. 8-, and 9.1-fold, respectively. In spite of that, total microbial biomass, bacteria, and G+ and G− bacteria were found to a greater extent in sewage sludge 100% than in PWS 100% after 4 months of composting. Conversely, fungi and actinobacteria were greater in PWS 100% (Figure 2). The most bacteria and, conversely, the least fungi were found in sludge 50% + PWS 50%, followed by sludge 25% + PWS 75%.

### Vermicomposting of Fresh and Precomposted Feedstock

#### Enzymes

During the first month of 100% fresh sewage sludge vermicomposting, there was a 65% decrease in the enzymatic activity of eight enzymes (Tables 2, 3). The enzymatic activity of alanine aminopeptidase decreased the most among enzymes during this period (by 87%). In contrast, acid phosphatase activity decreased by only 9%. The average activity of all monitored enzymes decreased in this variant by 95% after 4 months of vermicomposting. After 1 month of vermicomposting, no difference was found between the treatment with earthworms and without earthworms. However, after the 2^nd^ and 3^rd^ months, the enzymatic activity was greater in treatments with earthworms by 51 and 32%, respectively. After this time, no differences in average enzymatic activity were apparent. However, the situation was different for individual enzymes. PWS 100% was characterized by a significantly greater enzymatic activity in the earthworm treatments, especially in the case of β-D-glucosidase, acid phosphatase, lipase, chitinase, and leucin aminopeptidase. The least enzymatic activity was found at the vermicomposting end in mixtures, especially in sludge 75% + PWS 25%.

**TABLE 2:** Activities of β-D-glucosidase, acid phosphatase, arylsulphatase, and lipase during vermicomposting fresh feedstock.

	Sludge 100% without e.	Sludge 100% with e.	Sludge 75% + PWS 25% without e.	Sludge 75% + PWS 25% with e.	Sludge 50% + PWS 50% without e.	Sludge 50% + PWS 50% with e.	Sludge 25% + PWS 75% without e.	Sludge 25% + PWS 75% with e.	PWS 100% without e.	PWS 100% with e.
**β-D-glucosidase (μmol MUFG/h/g dw)**										
1 month	341 ± 94 aAB	248 ± 17 aAB	914 ± 196 aA	250 ± 71 aAB	139 ± 11 aAB	59 ± 26 aB	644 ± 202 aAB	779 ± 303 aA	118 ± 54 aAB	215 ± 34 aAB
2 months	26 ± 1 abA	25 ± 4 abA	50 ± 4 aAB	36 ± 12 abAB	140 ± 52 aB	44 ± 3 aAB	60 ± 16 abAB	38 ± 3 abAB	35 ± 11 bAB	57 ± 14 abAB
3 months	34 ± 8 abAB	14 ± 8 bAB	51 ± 13 aAB	31 ± 12 abAB	47 ± 16 aAB	4 ± 1 aA	39 ± 12 bAB	18 ± 8 bAB	61 ± 19 abB	27 ± 4 bAB
4 months	24 ± 5 bA	26 ± 8 abA	40 ± 24 aA	18 ± 8 bA	51 ± 18 aA	5 ± 1 aA	41 ± 14 abA	29 ± 5 abA	56 ± 1 abA	57 ± 27 abA
**Acid phosphatase (μmol MUFP/h/g dw)**										
1 month	1,734 ± 289 aAB	4,045 ± 1,107 aAB	3,111 ± 701 aAB	1,867 ± 628 aAB	1,413 ± 520 aAB	899 ± 208 aB	3,690 ± 1,403 aAB	7,065 ± 2,047 aA	970 ± 293 aB	1,295 ± 342 aAB
2 months	280 ± 56 bA	368 ± 146 abA	1,859 ± 86 abB	517 ± 65 abAB	1,319 ± 312 aAB	458 ± 41 abAB	749 ± 207 abAB	551 ± 83 aAB	893 ± 218 aAB	729 ± 190 abAB
3 months	385 ± 121 abAB	212 ± 41 bAB	378 ± 108 abAB	90 ± 19 bA	613 ± 152 aB	372 ± 103 abAB	421 ± 37 bAB	275 ± 96 aAB	235 ± 87 aAB	457 ± 112 abAB
4 months	423 ± 177 abA	243 ± 15 abA	331 ± 112 bA	209 ± 50 abA	572 ± 237 aA	317 ± 136 bA	516 ± 42 abA	254 ± 57 aA	280 ± 30 aA	269 ± 84 bA
**Arylsulphatase (μmol MUFS/h/g dw)**										
1 month	31 ± 5 aA	31 ± 3 aA	27 ± 12 aAB	19 ± 9 aAB	5 ± 2 aAB	0 ± 0 aB	13 ± 0 aAB	12 ± 6 aAB	10 ± 0 aAB	9 ± 0 aAB
2 months	3 ± 0 abAB	4 ± 0 bA	3 ± 1 aAB	3 ± 2 abAB	0 ± 0 bB	0 ± 0 aB	2 ± 1 bAB	1 ± 1 abAB	4 ± 0 bAB	3 ± 1 bAB
3 months	1 ± 0 bAB	5 ± 2 abAB	4 ± 3 aAB	4 ± 2 abAB	3 ± 1 abAB	5 ± 2 aAB	4 ± 3 abAB	0 ± 0 bA	7 ± 2 abB	4 ± 1 abAB
4 months	5 ± 3 abA	5 ± 0 abA	3 ± 2 aA	0 ± 0 bA	2 ± 1 abA	4 ± 3 aA	5 ± 3 abA	1 ± 1 abA	6 ± 2 abA	6 ± 1 abA
**Lipase (μmol MUFY/h/g dw)**										
1 month	3,375 ± 772 aAB	2,976 ± 352 aAB	4,153 ± 1,009 aAB	2,449 ± 780 aAB	5,372 ± 1,490 aB	3,262 ± 268 aAB	2,837 ± 624 aAB	3,115 ± 763 aAB	1,333 ± 375 aA	1,489 ± 914 aA
2 months	748 ± 113 abAB	648 ± 345 aAB	1,436 ± 275 abA	422 ± 118 aB	1,234 ± 263 abAB	830 ± 203 abAB	892 ± 148 abAB	830 ± 267 abAB	360 ± 155 aB	914 ± 197 aAB
3 months	396 ± 72 bA	477 ± 53 aA	615 ± 160 abA	582 ± 498 aA	405 ± 8 bA	527 ± 250 abA	325 ± 181 bA	213 ± 93 bA	404 ± 206 aA	440 ± 153 aA
4 months	716 ± 197 abA	515 ± 289 aA	490 ± 189 bA	382 ± 52 aA	452 ± 128 abA	371 ± 28 bA	439 ± 110 abA	386 ± 182 abA	426 ± 107 aA	445 ± 92 aA

*Values are means ± SD (n = 3). Different lowercase letters in a column indicate significant differences between months, while capital letters indicate significant differences among treatments (Kruskal–Wallis test, P ≤ 0.05).*

*dw, dry weight; PWS, pelletized wheat straw; e., earthworms; MUFG, 4-methylumbellyferyl-β-D-glucopyranoside; MUFP, 4-methylumbellyferyl-phosphate; MUFS, 4-methylumbellyferyl sulfate potassium salt; MUFY, 4-methylumbellyferyl-caprylate.*

**TABLE 3:** Activities of chitinase, cellobiohydrolase, alanine aminopeptidase, and leucine aminopeptidase during vermicomposting fresh feedstock.

	Sludge 100% without e.	Sludge 100% with e.	Sludge 75% + PWS 25% without e.	Sludge 75% + PWS 25% with e.	Sludge 50% + PWS 50% without e.	Sludge 50% + PWS 50% with e.	Sludge 25% + PWS 75% without e.	Sludge 25% + PWS 75% with e.	PWS 100% without e.	PWS 100% with e.
**Chitinase (μmol MUFN/h/g dw)**										
1 month	525 ± 90 aA	329 ± 7 aAB	391 ± 131 aAB	191 ± 21 aAB	243 ± 59 aAB	380 ± 64 aAB	297 ± 121 aAB	183 ± 30 aAB	59 ± 11 aB	57 ± 30 aB
2 months	42 ± 22 abAB	20 ± 12 aAB	42 ± 8 abAB	15 ± 7 abA	125 ± 30 abB	50 ± 4 abAB	83 ± 30 abAB	43 ± 8 abAB	17 ± 4 bAB	39 ± 17 aAB
3 months	29 ± 10 bAB	26 ± 10 aAB	27 ± 7 abAB	22 ± 10 abAB	41 ± 1 abA	20 ± 6 bAB	35 ± 16 bAB	11 ± 5 abB	33 ± 15 abAB	34 ± 3 aAB
4 months	60 ± 21 abA	20 ± 6 aAB	23 ± 4 bAB	13 ± 1 bAB	33 ± 4 bAB	29 ± 9 abAB	42 ± 19 abAB	10 ± 0 bB	30 ± 15 abAB	47 ± 9 aAB
**Cellobiohydrolase (μmol MUFC/h/g dw)**										
1 month	57 ± 21 aA	40 ± 6 aA	311 ± 46 aA	63 ± 21 aA	111 ± 29 aA	134 ± 22 aA	300 ± 168 aA	326 ± 1 aA	38 ± 22 aA	106 ± 26 aA
2 months	9 ± 5 abA	7 ± 5 aA	13 ± 5 abA	7 ± 3 abA	16 ± 7 abA	11 ± 0 abA	23 ± 6 abA	9 ± 6 abA	16 ± 2 abA	12 ± 0 aA
3 months	4 ± 3 bA	15 ± 9 aA	7 ± 3 abA	10 ± 6 abA	12 ± 3 abA	5 ± 2 abA	9 ± 4 bA	3 ± 2 bA	12 ± 2 bA	10 ± 2 aA
4 months	6 ± 3 abAB	6 ± 2 aAB	6 ± 2 bAB	3 ± 1 bA	6 ± 1 bAB	4 ± 3 bAB	12 ± 5 abAB	6 ± 3 abAB	35 ± 3 abB	10 ± 5 aAB
**Alanine aminopeptidase (μmol AMCA/h/g dw)**										
1 month	98 ± 2 aAB	151 ± 43 aA	143 ± 57 aAB	88 ± 21 aAB	27 ± 7 aB	32 ± 4 aAB	81 ± 10 aAB	89 ± 37 aAB	44 ± 1 aAB	28 ± 3 aB
2 months	34 ± 2 aAB	28 ± 12 aAB	58 ± 11 abAB	33 ± 14 abAB	42 ± 9 aAB	31 ± 1 abA	78 ± 4 aAB	47 ± 3 abAB	43 ± 5 aAB	82 ± 4 bB
3 months	28 ± 16 aA	25 ± 11 aA	39 ± 19 abA	27 ± 7 abA	41 ± 10 aA	18 ± 1 abA	25 ± 12 aA	15 ± 1 abA	38 ± 16 aA	44 ± 19 abA
4 months	25 ± 4 aAB	23 ± 4 aAB	22 ± 6 bAB	12 ± 7 bAB	10 ± 7 aAB	13 ± 2 bAB	20 ± 7 aAB	5 ± 1 bA	46 ± 2 aB	45 ± 3 abB
**Leucine aminopeptidase (μmol AMCL/h/g dw)**										
1 month	112 ± 14 aA	197 ± 65 aAB	147 ± 45 a	90 ± 14 aA	166 ± 15 aAB	286 ± 63 aAB	292 ± 58 aAB	419 ± 97 aB	194 ± 59 aAB	258 ± 88 aAB
2 months	91 ± 37 abAB	87 ± 22 abAB	66 ± 37 abAB	26 ± 11 abA	78 ± 4 abAB	224 ± 25 abAB	210 ± 60 aAB	237 ± 19 abA	55 ± 5 abAB	215 ± 64 aAB
3 months	38 ± 2 bAB	15 ± 9 bAB	23 ± 2 abAB	20 ± 8 abAB	25 ± 8 abAB	85 ± 37 abAB	12 ± 4 aA	12 ± 1 abA	47 ± 9 abAB	129 ± 7 aB
4 months	43 ± 5 abAB	32 ± 7 abAB	22 ± 1 bAB	9 ± 2 bA	9 ± 4 bA	21 ± 9 bAB	9 ± 4 aAB	11 ± 2 bAB	44 ± 7 bAB	114 ± 35 aB

*Values are means ± SD (n = 3). Different lowercase letters in a column indicate significant differences between months, while capital letters indicate significant differences among treatments (Kruskal–Wallis test, P ≤ 0.05).*

*dw, dry weight; PWS, pelletized wheat straw; e. = earthworms; MUFN, 4-methylumbellyferyl-N-acetylglucosaminide; MUFC, 4-methylumbellyferyl-N-cellobiopyranoside; AMCA, L-alanine-7-amido-4-methylcoumarin; AMCL, L-leucine-7-amido-4-methylcoumarin.*

**FIGURE 3:**
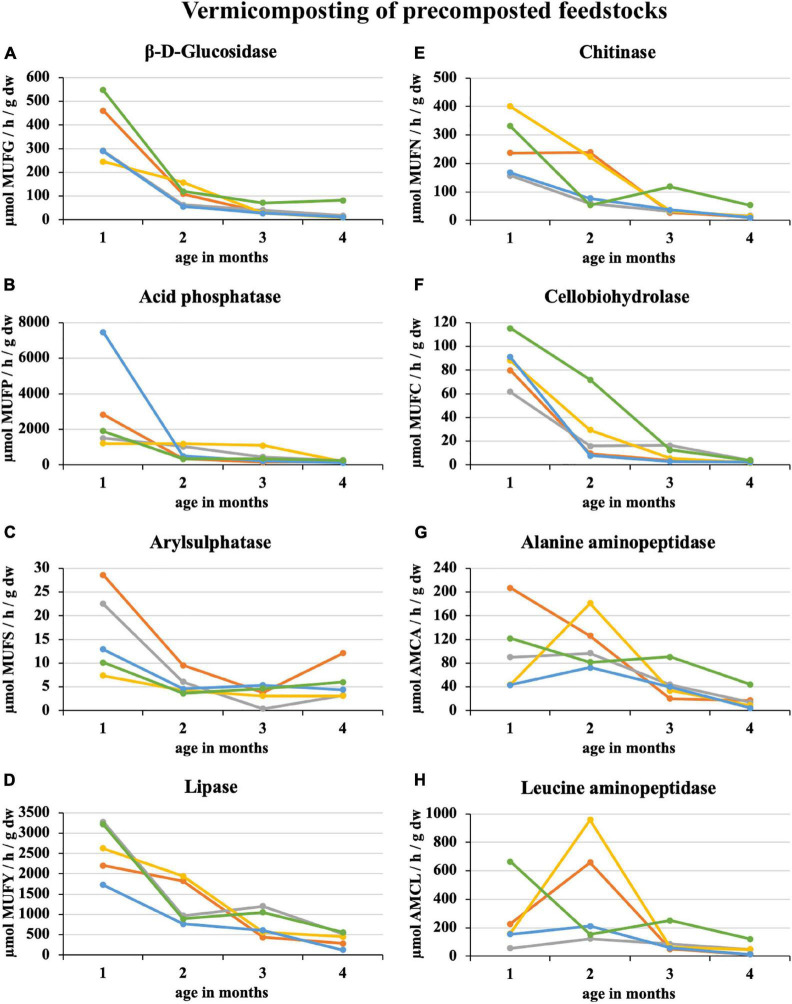
Course of enzymatic activity during vermicomposting of precomposted feedstock. 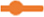, sludge 100% with earthworms; 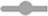, sludge 75% + PWS 25% with earthworms; 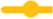, sludge 50% + PWS 50% with earthworms; 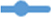, sludge 25% + PWS 75% with earthworms; 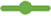, PWS 100% with earthworms; PWS, pelletized wheat straw; dw, dry weight; MUFG, 4-methylumbellyferyl-β-D-glucopyranoside; MUFP, 4-methylumbellyferyl-phosphate; MUFS, 4-methylumbellyferyl sulfate potassium salt; MUFY, 4-methylumbellyferyl-caprylate; MUFN, 4-methylumbellyferyl-N-acetylglucosaminide; MUFC, 4-methylumbellyferyl-N-cellobiopyranoside; AMCA, L-alanine-7-amido-4-methylcoumarin; AMCL, L-leucine-7-amido-4-methylcoumarin.

During 2 weeks of precomposting, the enzymatic activity of eight enzymes in 100% sewage sludge decreased by an average of 45%. A further decrease of 25% was recorded in the first month of subsequent vermicomposting (Figure 3). At the end of the 1^st^ month of vermicomposting, some enzyme values were greater than at the beginning (i.e., immediately after precomposting). Specifically, these enzymes were PWS (all enzymes), sludge 25% + PWS 75% (acid phosphatase, arylsulphatase, chitinase, cellobiohydrolase, alanine aminopeptidase, and leucin aminopeptidase), sludge 50% + PWS 50% (acid phosphatase, chitinase, alanine aminopeptidase, and leucin aminopeptidase), sludge 75% + PWS 25% (acid phosphatase), and sludge 100% (acid phosphatase and cellobiohydrolase). Thus, acid phosphatase increased in all treatments. Since the end of the 1^st^ month, enzymatic activity decreased (the same Figure 3). During vermicomposting of precomposted feedstocks, the enzymatic activity decreased. For most enzymes, it was significant after the 1^st^ month. As in the case of composting, alanin and leucin aminopeptidase, in some treatments (e.g., Sludge 50% + PWS 50%), increased after 1 month and decreased later. Of the monitored treatments, the greatest decrease (91%) was in the sludge 25% + PWS 75% treatment, followed by the sludge 100% treatment (by 89%), sludge 50% + PWS 50% (by 83%), sludge 25% + PWS 75% (by 79%), and PWS 100% (by 78%).

#### Earthworms

**FIGURE 4:**
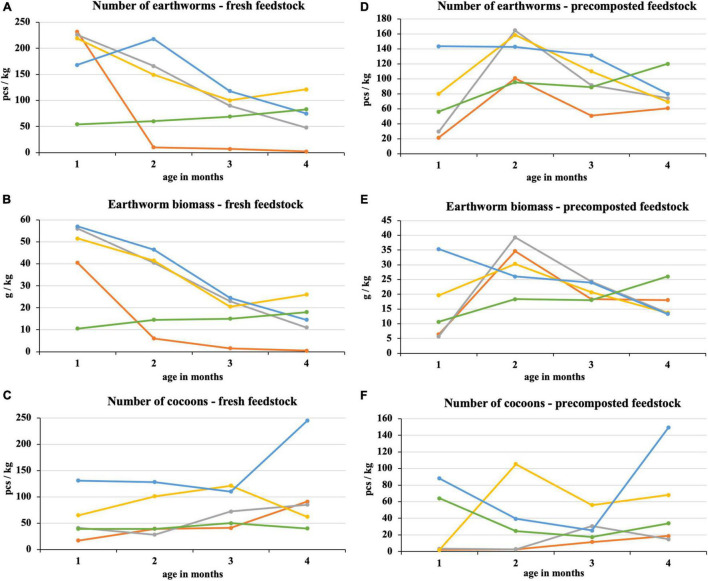
Course of number of earthworms, earthworm biomass, and number of cocoons in treatments with vermicomposting of fresh feedstock (**A–C**) and with vermicomposting of precomposted feedstock (**D–F**). 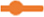, sludge 100% with earthworms; 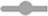, sludge 75% + PWS 25% with earthworms; 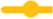, sludge 50% + PWS 50% with earthworms; 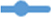, sludge 25% + PWS 75% with earthworms; 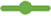, PWS 100% with earthworms; PWS, pelletized wheat straw.

After the first month of fresh feedstock vermicomposting, most earthworms were in 100% sewage sludge and mixtures with its high proportion (Figure 4). In these treatments, there was a subsequent decrease in earthworm number, which was directly proportional to the sewage sludge proportion. The difference between the process beginning and end was statistically significant in the sewage sludge 100% and sewage sludge 75% + PWS 25%. After the first month, the earthworm number in sludge 25% + PWS 75% increased (Figure 4A), but the earthworm biomass decreased (Figure 4B), which was due to the reduced weight of individual earthworm pieces. PWS 100% showed four times less earthworm number and five times less earthworm biomass than other treatments at the process beginning. During the following months, however, these parameters increased, and this treatment was included among the other mixed treatments. The earthworms decreased significantly in 100% sewage sludge, where only two pieces per kilogram were present at the end of vermicomposting. The cocoon number (Figure 4C) in the vermicomposted material increased during the process (at the beginning, 17–131 pieces/kg; in the end, 40–245 pieces/kg). Sewage sludge 25% + PWS 75% was characterized by the greatest number of loaded cocoons.

In the case of precomposted feedstock, the greatest number and earthworm biomass were found in three treatments with the greatest sludge proportion at the end of the 2^nd^ month of vermicomposting (Figures 4D,E). An approximately six-fold increase was found in the 75% sludge treatment. The earthworm number in the 25% sludge treatment was the greatest of all tested treatments at the process beginning (143 pieces/kg) and differed statistically from the 100% sludge treatment. It was the most stable treatment with a slight decrease. Conversely, PWS 100% showed a continuous increase in earthworm number and biomass. The earthworm number in this treatment varied statistically at the process beginning and in the end and reached the greatest values among all treatments (120 pieces/kg). PWS addition to the sludge and PWS itself caused an increase in the cocoon number in the 1^st^ month and at the process end (Figure 4F). The cocoon number in the 100% sludge and the 75% sludge treatments increased slightly in the 2^nd^ half of vermicomposting. Nevertheless, these treatments contained fewer cocoons at the end of vermicomposting compared to treatments with a greater PWS proportion.

#### Microorganisms

Similarly, as in the case of composting, fresh sewage sludge 100% differed from PWS 100% in the content and proportion of microorganism groups. During vermicomposting of 100% sewage sludge, total microbial biomass, fungi, bacteria, actinobacteria, and G+ and G− bacteria decreased and accounted for 9, 4, 9, 31, 13, and 5% of used feedstock, respectively. Conversely, the microorganism content increased in PWS 100% at the end, including total microbial biomass, fungi, bacteria, actinobacteria, and G+ and G− bacteria by 7. 4-, 1. 7-, 13. 4-, 10. 5-, 17. 6-, and 15.2-fold, respectively. After 4 months of vermicomposting, the earthworm treatments contained 60% of fungi, 80% of bacteria, 112% of actinobacteria, 86% of G+ bacteria, 73% of G− bacteria, and 71% of total microbial biomass compared to treatments without earthworms (Table 4). However, PWS 100% contained a greater number of microorganisms, except fungi, than the control treatment. Within treatments with and without earthworms, the greatest number of fungi, bacteria, actinobacteria, G+ bacteria, G− bacteria, and total microbial biomass was found in PWS 100% without earthworms, sludge 100% without earthworms, sludge 100% with earthworms, PWS 100% with earthworms, PWS 100% with earthworms, and sludge 75% + PWS 25% without earthworms, respectively.

**TABLE 4:** Content of microorganisms after 4 months of vermicomposting fresh feedstock.

	Sludge 100% without e.	Sludge 100% with e.	Sludge 75% + PWS 25% without e.	Sludge 75% + PWS 25% with e.	Sludge 50% + PWS 50% without e.	Sludge 50% + PWS 50% with e.	Sludge 25% + PWS 75% without e.	Sludge 25% + PWS 75% with e.	PWS 100% without e.	PWS 100% with e.
Fungi (μg PLFA/g dw)	12 ± 8 a	6 ± 0 ab	13 ± 5 ab	6 ± 1 ac	23 ± 5 ab	11 ± 1 ab	28 ± 13 ab	21 ± 7 ab	48 ± 16 b	41 ± 3 bc
Bacteria (μg PLFA/g dw)	514 ± 258 ab	272 ± 8 ab	430 ± 248 ab	194 ± 14 a	364 ± 128 ab	180 ± 23 a	331 ± 116 ab	252 ± 14 ab	422 ± 18 ab	741 ± 18 b
Actinobacteria (μg PLFA/g dw)	8 ± 0 ab	11 ± 1 a	8 ± 1 ab	9 ± 0 ab	7 ± 0 ab	6 ± 0 b	7 ± 1 ab	7 ± 0 ab	7 ± 2 ab	10 ± 1 a
G+ (μg PLFA/g dw)	215 ± 77 ab	150 ± 3 ab	180 ± 83 ab	97 ± 10 a	167 ± 50 ab	87 ± 23 a	160 ± 59 ab	115 ± 8 ab	193 ± 13 ab	353 ± 12 b
G− (μg PLFA/g dw)	254 ± 166 ab	85 ± 6 ab	208 ± 152 a	65 ± 1 ab	160 ± 77 ab	74 ± 6 a	135 ± 54 ab	109 ± 7 ab	193 ± 4 ab	335 ± 8 b
Total microbial biomass (μg PLFA/g dw)	671 ± 319 ab	362 ± 14 ab	1,517 ± 1,083 a	263 ± 16 ab	479 ± 147 ab	233 ± 18 b	446 ± 155 ab	339 ± 28 ab	566 ± 12 ab	898 ± 16 a

*Values are means ± SD (n = 3). Different letters in a column indicate significant differences between treatments (Kruskal–Wallis test, P ≤ 0.05). dw, dry weight; PWS, pelletized wheat straw; e., earthworms; PLFA, phospholipid fatty acids.*

**FIGURE 5:**
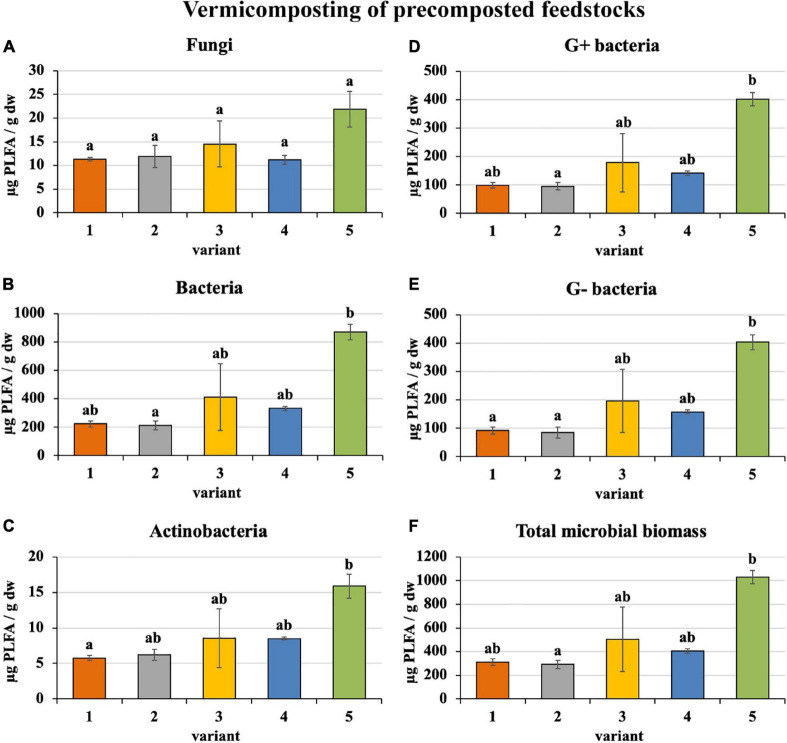
Content of microorganisms after vermicomposting precomposted feedstock. Treatments: (1) sludge 100% with earthworms, (2) sludge 75% + PWS 25% with earthworms, (3) sludge 50% + PWS 50% with earthworms, (4) sludge 25% + PWS 75% with earthworms, and (5) PWS 100% with earthworms. Values are means ± SD (*n* = 3). Different letters indicate significant differences among treatments (Kruskal–Wallis test, *P* ≤ 0.05). PWS, pelletized wheat straw; PLFA, phospholipid fatty acids; dw, dry weight.

Microorganism content in precomposted treatments at the process end was slightly greater (bacteria—410 μg/g, actinobacteria—9 μg/g, G+ bacteria—183 μg/g, G− bacteria—187 μg/g, and total microbial biomass—508 μg/g), as shown in Figure 5, than in the case of freshly used feedstocks described above (bacteria—328 μg/g, actinobacteria—8 μg/g, G+ bacteria—160 μg/g, G− bacteria—134 μg/g, and total microbial biomass—419 μg/g). Fungi were exceptions (in precomposted treatments—14 μg/g and in treatments with fresh feedstocks—17 μg/g). The greatest content of individual microorganism groups were unequivocally found in PWS 100%, followed by sludge 50% + PWS 50%, sludge 25% + PWS 75%, and almost identically sludge 100% and sludge 75% + PWS 25%.

## Discussion

During sludge composting and treatments with its addition, there was a significant and permanent decrease in β-D-glucosidase activity. In the case of straw pellets, there was a reduced but constant enzyme activity because of slow degradation. Plant residues primarily composed of cellulose, hemicellulose, or lignin are difficult to biodegrade and increase composting time, which is associated with longer enzymatic activity (Hemati et al., 2021). Estrella-González et al. (2019) determined profiles of β-glucosidase, amylase, cellulase, and xylanase activities during large-scale composting of vegetable waste, urban solid waste, sewage sludges, agrifood waste, and olive oil mill wastewater. The results revealed very different profiles. The evolution profiles of the enzymes involved in the degradation of lignocellulosic fractions in sewage sludges coincided with greater biodegradation rates of these fractions at the process end. The β-D-glucosidase decrease in sewage sludge copied the trend we found. Unfortunately, it is not possible to compare specific values because we utilized the more effective and modern fluorescence method, and the previous study used classical colorimetric estimation of p-nitrophenol released by p-nitrophenyl-β-D-glucopiranoside hydrolysis.

The greatest acid phosphatase activity was found at composting beginning in 100% sewage sludge, which was because of the high phosphorus content in this feedstock. This statement is confirmed by Albrecht et al. (2010) who co-composted green waste and sewage sludge for 5 months. Acid phosphatase activities were high during the first month of composting and then declined. Large nutrient quantities stimulated the growth of total aerobic bacteria and subsequent phosphatase activity.

Ma et al. (2019) found that arylsulphatase content in sewage sludge (75%) + sawdust (25%) initially increased and subsequently decreased from the initial rate of 10.05 to 15.51 μmol/(h × g) at the end of composting. By contrast, the arylsulphatase content in sewage sludge (75%) + sawdust (12.5%) + matured compost (12.5%) decreased continuously. The arylsulphatase content in the matured compost treatment was significantly greater than in the treatment without matured compost at the mesophilic phase. After entering the thermophilic phase, the arylsulphatase content in sewage sludge (75%) + sawdust (12.5%) + matured compost (12.5%) was significantly greater than that without matured compost, and this difference was maintained until the composting end (*P* < 0.05). Therefore, the arylsulphatase content increased during the mesophilic phase and decreased during the cooling phase when the matured compost was added. In our experiment, the arysulphatase activity increased after the 2^nd^ month of composting in the treatments with 50 and 75% straw pellets and in the case of the pellets themselves in the 2^nd^ half of the process. This may be because of the gradual release of organic sulfur from straw with the degradation of cellulose and other refractory organic matter, resulting in increased arylsulphatase content (Tejada et al., 2006).

Gea et al. (2007) co-composted sewage sludge with 30 and 50% fat addition. Lipase from a thermophilic composting environment showed a high stability for mesophilic temperature values and slightly alkaline pH values. The maximum lipolytic activity was observed at the thermophilic phase, which is consistent with our findings where the greatest lipase activity was during the first month of composting and then declined. Lipase activity in this period ranged from 3,300 to 5,000 μmol MUFY h^–1^ g^–1^ dw, with the exception of sludge 25% + PWS 75% (9360 μmol MUFY h^–1^ g^–1^ dw), and decreased to 1,000 μmol MUFY/h/g dw at composting end.

Chitinase activity varied greatly among treatments at the beginning of composting. The values were directly proportional to the proportion of sewage sludge. Poulsen et al. (2008) reported that chitinase activity was much greater in household waste compost (3.97 μmol 4 MU/h × g dry matter) than in garden/park compost (0.46 μmol 4 MU/h × g dry matter). Chitinase genes are found in a range of microorganisms, in particular actinomycetes, and in fungi (Krsek and Wellington, 2001). These organisms were apparently present and active to a greater extent during sludge composting and mixtures with a great proportion of this feedstock.

Cellobiohydrolase activity strongly correlated with β-D-glucosidase because they participated in carbonaceous substance decomposition, especially saccharides such as cellulose and cellobiose. The greatest activity of both enzymes was recorded in sludge 25% + PWS 75%, especially in the first half of the composting. The sludge significantly supported and accelerated microorganism development and thus the decomposition of the above-mentioned substances in straw pellets. The activity of both enzymes was significantly greater compared to the straw pellets itself.

The profile of alanine and leucine aminopeptidase was different from those of other enzymes during composting. After 2 months of composting, there was an increase in enzymatic activity. This increase was greatest at 75 and 100% straw pellets. Thus, enzyme activity must have been related to terminal nitrogen release from alanine and leucine found in straw. For a detailed evaluation, it would be necessary to establish further experiments and also to monitor the amino acid content.

Similar to composting, the greatest enzymatic activity decrease occurred in the first half of vermicomposting. Straw is as an important bulking agent because it matures compost and vermicompost faster as evidenced by decreased enzymatic activities. A great proportion of straw pellets proved successful in vermicomposting coffee grounds (Hanc et al., 2021). The greatest earthworm number and biomass were in the treatment with 75% straw pellets. The experiment was operated in a system with continuous earthworm feeding. The following enzymatic activities were detected in the oldest layer (6 months) of this treatment: β-D-glucosidase—1,194 μmol MUFG/h/g dw, acid phosphatase—1,894 μmol MUFP/h/g dw, arylsulphatase—64 μmol MUFS/h/g dw, lipase—3,924 μmol MUFY/h/g dw, chitinase—207 μmol MUFN/h/g dw, cellobiohydrolase—105 μmol MUFC/h/g dw, alanine aminopeptidase—46 μmol AMCA/h/g dw, and leucine aminopeptidase—55 μmol AMCL/h/g dw. These values were greater than in the current study, which was due to a different processed raw material and technological procedure. During vermicomposting of sewage sludge and coffee grounds, lesser enzymatic activities were found in treatments with earthworms than those without earthworms. Similarly, Domínguez and Gómez-Brandón (2012) reported that earthworm activity greatly reduced protease and cellulase enzyme activities in comparison with the control. These findings are in agreement with the microbial activity data, which reinforces that a greater degree of stability was reached after the active vermicomposting phase. This is also related to the microbiota composition changes in the intestines of earthworms and vermicompost. Domínguez et al. (2021) utilized 16S and ITS rRNA high-throughput sequencing to characterize bacterial and fungal community composition and structure during the gut-associated processes (GAP) and cast-associated processes of sewage sludge vermicomposting. The bacterial and fungal communities of earthworm casts were mainly composed of microbial taxa not found in the sewage sludge. Thus, most of the bacterial (96%) and fungal (91%) taxa in the sewage sludge were eliminated during vermicomposting, mainly through the GAP. Less microbial biomass, mainly fungi, was found in the earthworm treatments, which is consistent with the claim that fungi are a valuable food source (Zhang et al., 2000). Hřebečková et al. (2019) compared the enzymatic activity in three aged vermicompost types (from household biowaste, malt house sludge mixed with agricultural waste, and grape marc). The vermicomposting was conducted in large-scale heap systems with continuous earthworm feeding, which is applicable in practice. The greatest hydrolytic enzyme activity occurred in the vermicomposting process with household biowaste.

It is necessary to approach enzymatic activity values during composting and vermicomposting with caution. The enzymatic activity amount depends mainly on the feedstock types used as well as the maturity phase and the technological process used. In the case of vermicomposting, earthworm density and activity are important.

Further research should be focused on determining the effectiveness of sewage sludge composting and vermicomposting using other bulking agents. Furthermore, it would be appropriate to verify the materials used from the first half of the composting and vermicomposting period, when enzymatic activity was great. It would be interesting to use straw pellets, which are characterized by purity and whose enzymatic activity remained at greater values for a longer time period, both during composting and vermicomposting compared to other treatments. This could be used as a cheap way, for example, for the acceleration of biowaste decomposition and isolation of enzymes for the production of enzymatic preparations or for the revitalization of biologically inactive or contaminated soil.

## Data Availability Statement

The raw data supporting the conclusions of this article will be made available by the authors, without undue reservation.

## Author Contributions

AH contributed to conceptualization, methodology, investigation, writing, visualization, funding acquisition, formal analysis, and project administration. BD contributed to experiments, analyses, and data curation. TH contributed to data curation and formal analysis. All authors contributed to the article and approved the submitted version.

## Conflict of Interest

The authors declare that the research was conducted in the absence of any commercial or financial relationships that could be construed as a potential conflict of interest.

## Publisher’s Note

All claims expressed in this article are solely those of the authors and do not necessarily represent those of their affiliated organizations, or those of the publisher, the editors and the reviewers. Any product that may be evaluated in this article, or claim that may be made by its manufacturer, is not guaranteed or endorsed by the publisher.
